# The effects of negative life events on college students’ problematic online gaming use: a chain-mediated model of boredom proneness regulation

**DOI:** 10.3389/fpsyg.2024.1426559

**Published:** 2024-12-06

**Authors:** Zhenyu Zhao, Mengmeng Zhao, Ruixin Wang, Huiru Pan, Lina Li, Hongge Luo

**Affiliations:** School of Psychology and Mental Health, North China University of Science and Technology, Tangshan, China

**Keywords:** negative life events, basic psychological needs, anxiety, problematic online gaming use, boredom proneness

## Abstract

The study investigated the impact of negative life events on college students’ problematic online gaming use, as well as the mediating role of basic psychological needs and anxiety, and the moderating effect of boredom proneness. A total of 1,102 college students were surveyed by using the Adolescent Negative Life Events Scale, Basic Psychological Needs Scale, GAD-7, DSM-5, and Boredom Proneness Scale. From the sample, 881 participants with experience in online gaming were selected for the study. The results showed that: (1) Controlling for gender and grade, negative life events significantly and positively predicted problematic online gaming use. (2) Basic psychological needs and anxiety played a mediating role in the relationship between negative life events and problematic online gaming use. (3) Boredom proneness significantly moderated the first half of the model. Basic psychological needs and anxiety mediate the relationship between negative life events and college students’ problematic online gaming use. Moreover, under conditions of low boredom proneness, the independent mediating effect of basic psychological needs and the mediating effect of basic psychological needs and anxiety are enhanced, while the independent mediating effect of anxiety is weakened.

## Introduction

Internet gaming is a global recreational activity that plays an important role in the social and leisure activities of young people. However, excessive addiction to internet gaming can negatively impact the psychological well-being and academic performance of adolescents, and even lead to mental disorders and psychological illnesses (Yen et al., 2017). *The Diagnostic and Statistical Manual of Mental Disorders* (DSM-5) categorizes Internet Gaming Disorder (IGD) as a behavioral disorder (Tullett-Prado et al., 2021), noting its many similarities to substance use disorders in physiological, psychological, and social aspects (Jin et al., 2019; Pontes and Griffiths, 2014). Furthermore, the prevalence of internet gaming disorder among college students is 11%, which is higher compared to other student populations (Shao et al., 2018). Current empirical research has discussed college students’ problematic online gaming use from various perspectives such as individual psychology and living environment. The I-PACE model (the interaction of person-affect-cognition-execution model) integrates a wealth of existing empirical research and theoretical models, suggesting that internet addiction is the result of interactions between triggering, moderating, and mediating variables (Brand et al., 2016). The I-PACE model, which focuses on the interaction between individual, emotional, cognitive, and executive aspects, particularly highlights the influence of environmental factors on internet use behavior (Brand et al., 2016). Within this model, the P component, representing personality traits, is a foundational variable and serves as a precipitating factor for excessive internet use. The A and C components are determinants that serve as moderating and mediating factors influencing the decision to use the internet. The interaction between these components is crucial for leading to addictive behavior, as they collectively contribute to the development of internet addiction. However, this model is currently underutilized in the field of problematic online gaming use research and lacks exploration of interaction patterns among various factors, making it somewhat disconnected from existing theories (Zhang et al., 2022). Therefore, this study is based on the I-PACE model and combines it with other theoretical models to examine the psychological mechanisms of problematic online gaming use in college students, aiming to provide a scientific basis for clinical interventions.

### Impact of negative life events on problematic online gaming use

Negative life events refer to significant changes that individuals experience, forcing them to make stress responses to adapt to the changes. They have adverse effects on the physical and mental health of individuals (Rabkin and Struening, 1976). According to the general strain theory, the stress generated by negative life events can lead to negative emotions, prompting individuals to engage in maladaptive or addictive behaviors to alleviate these emotions (Robert, 2012). In the I-PACE model, it is posited that stress arises from an individual’s perception of the external environment. Concurrently, the bioecological model emphasizes that psychology and behavior are products of the interaction between individual development and the environment, with a particular focus on the impact of the environment. Adverse life events are considered the concrete manifestation of stressful situations (Fang, 2009). Adolescents are in a transitional period from childhood to adulthood, during which they face pressures from various aspects such as school, family, and society, including academic, financial, and emotional pressures. Internet gaming serves as an important outlet for them to relieve stress (Huang et al., 2017). Therefore, the first research hypothesis proposed in this study is that: negative life events can positively predict problematic online gaming use.

### Chain mediating role of basic psychological needs and anxiety

According to Self-Determination Theory, there are three fundamental psychological needs: the need for autonomy, competence, and relatedness. When these needs are met, individuals tend to develop positively. Conversely, when these needs are obstructed, an individual’s psychological and physical development may be impeded, potentially leading to functional impairments (Song and Zhao, 2021). Researches have found that the more negative life events an individual experiences, the lower the degree of satisfaction of their psychological needs (Wu et al., 2020). A study indicates that exposure to stressful life events significantly increases an individual’s risk of suicide, with basic psychological needs playing a crucial role in this process (Rowe et al., 2013). This suggests that basic psychological needs are an extremely important psychological resource when individuals face negative life events, offering a beneficial intervention for improving negative emotions. During times of severe or frequent stressful life events, it becomes difficult for individuals to satisfy their basic psychological needs, leading them to redirect their focus to other activities in an attempt to fulfill these needs (Yu et al., 2012; Van den Broeck et al., 2008). Based on the Use-Satisfaction Model of problematic internet use, individuals can fulfill psychological needs in the internet world that may not be met in reality, leading to increased reliance on the internet and the development of problematic internet use (Ko et al., 2005). Therefore, we hypothesize that basic psychological needs may serve as a mediator in the impact of negative life events on internet gaming disorder.

Life events have a profound impact on the mental health of college students, with studies showing that they are one of the direct factors affecting anxiety in adolescents (Huang Q. et al., 2021; Huang S. H. et al., 2021). The Vulnerability-Stress Model posits that certain dispositions, such as maladaptive personality traits and cognitive patterns, when activated by specific environmental factors, increase the risk of anxiety (Riskind and Alloy, 2006). Individuals with neurotic tendencies or those frequently in a state of anxiety are more prone to developing problematic online gaming use (Mehroof and Griffiths, 2010). Thus, we hypothesize that anxiety may act as a mediator in the influence of negative life events on internet gaming disorder.

Research demonstrates that when an individual’s basic psychological needs are unmet, their level of anxiety significantly increases (Przybylski et al., 2013). We believe that when individuals encounter negative events in life, family, or academics, their basic psychological needs may not be fulfilled, leading to feelings of anxiety. To alleviate this anxiety, individuals may be more inclined to indulge in internet gaming, using it as a means to mitigate their anxiety and fulfill certain psychological needs. There is a scarcity of research that considers the combined effects of basic psychological needs and anxiety when examining the impact of negative life events on internet gaming disorder.

Therefore, we propose Hypothesis 2: Basic psychological needs and anxiety exert a serial mediating role in the influence of negative life events on internet gaming disorder.

### Moderating effect of boredom proneness

Boredom proneness is a negative and relatively stable personality trait that can lead to an individual experiencing a heightened sense of negativity over time, such as feelings of loneliness, anxiety, and a lack of meaning (Huang et al., 2020). Chen (2016) posits that individuals with high levels of boredom proneness, due to their excessive introspection, struggle to derive joy and fulfillment from real-life experiences, resulting in inner turmoil and depression, thereby making them more susceptible to negative emotions such as anxiety and depression. Furthermore, the higher the degree of unmet basic psychological needs in adolescents, the more likely they are to experience boredom (Van Hooff and Van Hooft, 2017). According to Bronfenbrenner and Morris's (2006) Bioecological Model, individuals with varying characteristics often exhibit different adaptive outcomes when confronted with stressful situations. College students with high levels of boredom proneness are more likely to become engrossed in the novelty and excitement offered by internet gaming, which can lead to impairments in their physiological, psychological, and social functioning (Huang Q. et al., 2021; Huang S. H. et al., 2021). Therefore, we propose that the relationship between negative life events, anxiety, basic psychological needs, and problematic online gaming use is influenced by boredom proneness. Boredom proneness may serve as a potential moderating variable in the impact of negative life events on these three factors. There is a dearth of research examining boredom proneness as a moderating variable in the effects of negative life events on problematic online gaming use, basic psychological needs, and anxiety. Consequently, this study puts forth Hypothesis 3: The influence of negative life events on basic psychological needs, anxiety, and problematic online gaming use is moderated by boredom proneness. Specifically, when college students with high levels of boredom proneness encounter negative life events, they are likely to have a lower fulfillment of their basic psychological needs, higher levels of anxiety, and a greater risk of problematic online gaming use.

Environmental factors and related variables are significant predictors of internet addiction, the development of addictive behaviors occurs through the interaction between individual propensity variables and environmental factors. An individual’s perception of external situational factors may trigger cognitive and emotional responses, leading to experiences of satisfaction and compensation, which may subsequently lead to addictive phenomena (Zhou et al., 2025). The tendency towards boredom, as a core personality trait of an individual, is likely an important moderating variable. Early negative experiences (such as childhood abuse) or currently stressful life events are significantly associated with internet addiction (Li et al., 2020). Individuals with a high tendency towards boredom, when faced with negative life events, due to fewer external connections and an inability to effectively seek external support, are more likely to experience unmet psychological needs, leading to negative slacking, escape from reality, and engaging in risky behaviors such as indulging in online games; or they may seek satisfaction of psychological needs through online gaming (Wang J. P. et al., 2020). Moreover, individuals with a high tendency towards boredom, due to their greater focus on internal characteristics, are much more likely to experience anxiety when their basic psychological needs are not met. This strong internal sense of dissatisfaction prompts individuals to take action to change their state of boredom. At this point, online gaming, an activity that consumes little cognitive and emotional resources and provides high feedback, is very likely to become the optimal choice for an individual (Yu and Zhang, 2021).

### The present study

The I-PACE model suggests that the stress people experience due to negative events is subjectively perceived, and the subsequent cognitions and emotions affect the individual’s coping methods, increasing the risk of developing internet use disorders. Self-determination theory posits that psychological needs are an extremely important resource when individuals face stressful situations, enabling them to cope with stress in a positive manner. The bioecological model suggests that individuals with different characteristics will adopt different methods to cope with stress, and the vulnerability-stress model also posits that personality traits with weaker stress resistance are more likely to generate anxiety and other negative emotions when faced with stress. The personality characteristic of boredom proneness, marked by low arousal and low agency, makes it difficult to satisfy psychological needs when confronted with negative life events, leading more readily to anxiety and other negative emotions. If online gaming can provide a temporary escape from stressful situations and compensate for the missing psychological needs, alleviating anxiety, then the behavior of using online games is reinforced. In the early stages of problematic online game use, satisfaction is the main driving force behind changes in emotional and cognitive responses to internet addiction-related stimuli, and as this process continues, the compensation effect further increases (Brand et al., 2016).

**Figure 1:**
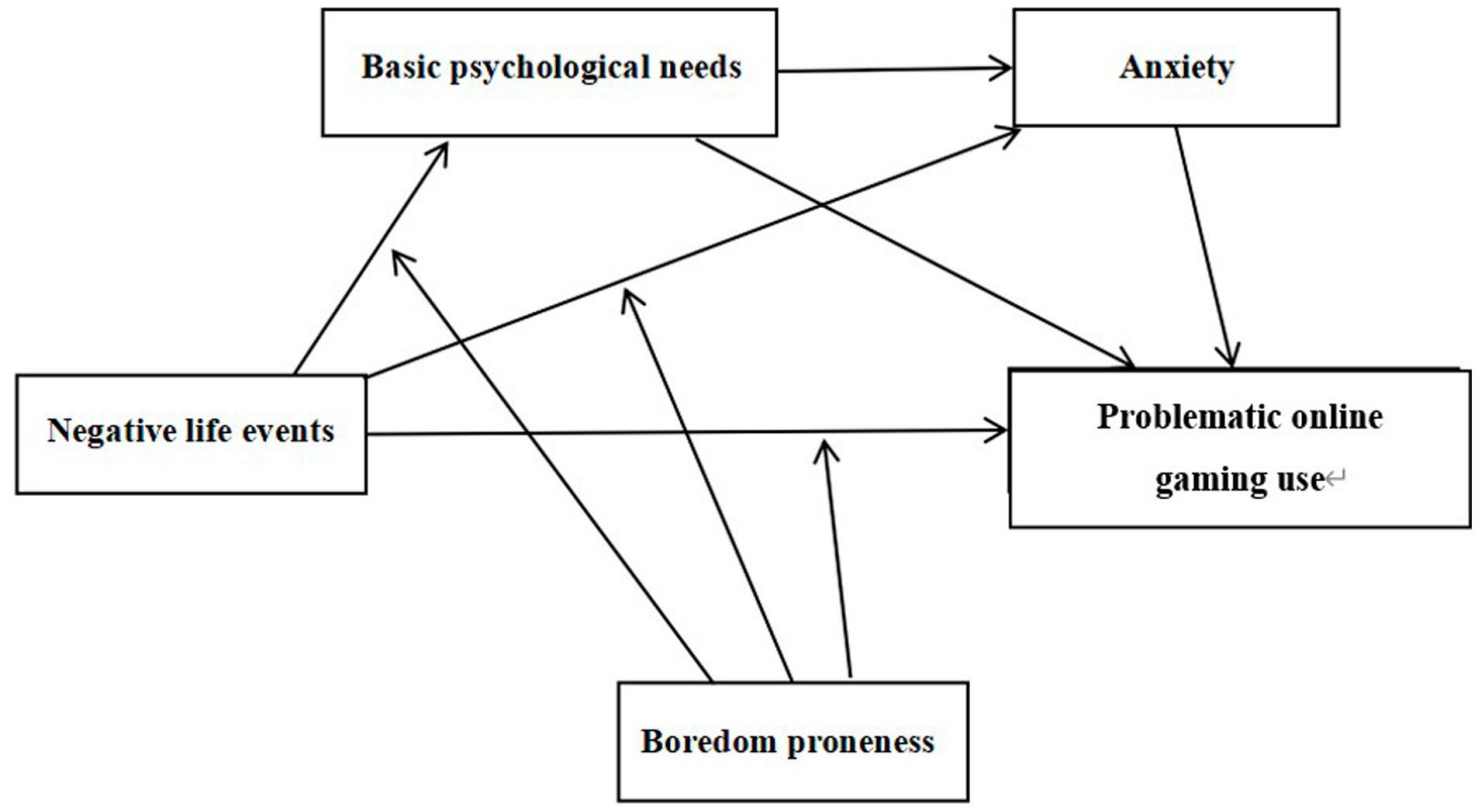
Theoretical model.

In summary, this study, grounded in the I-PACE model and informed by Self-Determination Theory, the Vulnerability-Stress Model, and Bronfenbrenner’s Bioecological Model, aims to construct a moderated serial mediation model. This model elucidates both the direct and indirect relationships between negative life events and problematic online gaming use, as well as the moderating effect of boredom proneness. The theoretical framework of the study is depicted in Figure 1.

## Method

### Participants

The sample size required for this study was calculated using the following formula, where N represents the required sample size, 1.96is the critical value from the standard normal distribution, corresponding to a 95% confidence level, p is the anticipated population proportion, for which we took the most conservative estimate of 0.5, and e is the maximum allowable margin of error desired by the researchers, which in this study was set at 5%. Ultimately, we determined the minimum sample size to be 384. Data was collected from 1,115 students across four grades (freshman to senior) at a certain university using the Wen juan xing (Questionnaire Star) platform. A total of 1,102 valid questionnaires were retrieved, yielding an effectiveness rate of 98.88%. Students with experience in online gaming were filtered, totaling 881 participants, who spent an average of 2.44 ± 2.02 h per day gaming. Among them, there were 457 females (60.1%) and 424 males (39.9%), with ages ranging from 17 to 23 years (mean age 18.86 ± 1.35).


$$N=\frac{{Z^{2}}^{*}p\left(1-p\right)}{e^{2}}$$

### Measures

#### Negative life events

The study employed the Adolescent Self-rating Life Events Checklist (ASLEC) (Xin and Yao, 2015). This questionnaire encompasses five factors: punishment received, loss, interpersonal stress, academic pressure, and adaptation issues, comprising a total of 26 items. The scale operates on a 6-point scoring system, ranging from 0 (no impact) to 5 (extremely severe), with higher scores indicating a greater impact of negative life events. The scale demonstrated a Cronbach’s *α* coefficient of 0.92 for internal consistency, a test–retest reliability of 0.73, and a split-half reliability of 0.85. In the context of this study, the Cronbach’s α coefficient for the scale was calculated to be 0.98.

#### Basic psychological needs

The study utilized the Basic Psychological Needs Satisfaction Scale (Deci and Ryan, 2000), which consists of 21 items, including nine reverse-scored items, categorized into three dimensions: autonomy, relatedness, and competence. The scale is rated on a 7-point scale, ranging from 1 (does not apply at all) to 7 (fully applies), with an average score for all items indicating the level of satisfaction of basic psychological needs; higher scores suggest a better state of satisfaction. In this study, the Cronbach’s *α* coefficient for the scale was 0.88.

#### Anxiety

The study employed the Generalized Anxiety Disorder-7 (GAD-7) scale (Spitzer et al., 2006), which is recognized for its high reliability and validity in screening for anxiety symptoms. The GAD-7 comprises seven items, each rated on a 4-point scale from 0 (not at all) to 3 (nearly every day), with the total score serving as the primary metric. Higher scores are indicative of a more severe level of anxiety. The scoring categorizes anxiety as follows: 0–4 points for no anxiety or anxiety not of clinical significance; 5–9 points for mild anxiety; 10–14 points for moderate anxiety; and 15 or more points for severe anxiety. In the context of this study, the Cronbach’s *α* coefficient for the scale was calculated to be 0.97.

Problematic online gaming use. The study utilized the criteria from the Diagnostic and Statistical Manual for Mental Disorders, Fifth Edition (DSM-5) questionnaire (Petry et al., 2014), to measure internet gaming disorder. The assessment is based on nine diagnostic criteria, employing a binary scoring system with options labeled ‘yes’ (scored as 1) and ‘no’ (scored as 0). A total score of 5 or higher is indicative of problematic online gaming use. In the context of this study, the Cronbach’s *α* coefficient for the scale was calculated to be 0.84.

#### Boredom proneness

The study employed the College Student Boredom Proneness Questionnaire (Huang et al., 2010), consisting of 30 items across two dimensions: internal stimulation and external stimulation. The external stimulation dimension encompasses four factors: constraint, monotony, loneliness, and tension, while the internal stimulation dimension includes two factors: self-control and creativity. The questionnaire utilizes a 7-point Likert scale (ranging from 1, “completely disagree,” to 7, “completely agree”), with reverse scoring applied to the internal stimulation dimension. A higher overall average score indicates a higher level of boredom proneness. The questionnaire boasts a Cronbach’s α coefficient of 0.93, and in the context of this study, the Cronbach’s α coefficient was found to be 0.92.

#### Statistical approach

Common method bias, correlation analysis, Independent Samples t-Test, One-Way ANOVA and hierarchical regression analysis were conducted using SPSS 23.0. Chain mediating effects were examined using Model 6 within the PROCESS microprogram in SPSS, while Model 85 was utilized for the assessment of moderating effects.

## Results

### Common method bias analysis

Common Method Bias was assessed using Harman’s single-factor test, where all items from the scales in this study were subjected to an unrotated exploratory factor analysis. The results indicated the presence of 11 factors with eigenvalues greater than one, with the first factor accounting for 27.974% of the variance, which is less than 40% (Zhou and Long, 2004). This suggests that there is no significant common method bias in the current study.

### Correlation analysis

Correlation analysis results indicate a significant positive correlation between negative life events in adolescents and problematic online gaming use, boredom proneness, and anxiety, while a significant negative correlation exists with basic psychological needs. Problematic online gaming use is significantly negatively correlated with basic psychological needs and positively correlated with boredom proneness and anxiety. Basic psychological needs are negatively correlated with both boredom proneness and anxiety, and these latter two factors are significantly positively correlated with each other. Gender is significantly positively correlated with negative life events, problematic online gaming use, and anxiety, and negatively correlated with basic psychological needs. Grade level is significantly positively correlated with negative life events and anxiety, and negatively correlated with basic psychological needs. For detailed information, refer to Table 1.

**Table 1:** Results of correlation analysis.

	*M ± SD*	Gender	Grade	NLE	IGA	BPN	BP	Anxiety
Gender	–	–						
Grade	–	–	–					
NLE	42.73 ± 29.49	0.11^**^	0.11^**^	1				
POGU	1.74 ± 2.30	0.19^***^	−0.01	0.26^***^	1			
BPN	86.54 ± 15.11	−0.13^***^	−0.10**	−0.36^***^	−0.46^***^	1		
BP	105.00 ± 26.90	−0.01	0.02	0.36^***^	0.46^***^	−0.68^***^	1	
Anxiety	4.99 ± 5.18	0.08^*^	0.13^***^	0.56^***^	0.30^***^	−0.46^***^	0.46^***^	1

*p < 0.05, **P < 0.01, ***P < 0.001 (the same applies below). NLE, Negative life events; POGU, problematic online gaming use; BPN, Basic psychological needs; BP, Boredom proneness.

### Examination of differences in problematic online gaming use by gender and grade

The Independent Samples *t*-Test was utilized to assess whether there were differences in problematic online gaming use between genders, with the results presented in Table 2. The statistical findings indicate a significant difference in problematic online gaming use among different genders (*t* = −6.15, *p* < 0.001), suggesting that males exhibit significantly higher levels of problematic online gaming use compared to females. A One-Way ANOVA was conducted to examine the differences in problematic online gaming use across grade levels, with the results depicted in Table 3. The statistical results suggest no significant differences in problematic online gaming use among different grade levels, *F*_(3,877)_ = 1.24, *p* = 0.295.

**Table 2:** Examination of gender differences in problematic online gaming use.

Gender	M	SD	*t*	*p*
Male	1.29	1.89	−6.15	<0.001
Female	2.23	2.58		

**Table 3:** Examination of grade differences in problematic online gaming use.

Grade	*M*	SD	df	*F*	*p*
1	1.68	2.12	3	1.24	0.295
2	1.67	2.36			
3	1.95	2.60			
4	0.88	1.13			

### The impact of negative life events, basic psychological needs, and anxiety on problematic online gaming use

Correlation analysis revealed a significant association between gender and problematic online gaming use. Consequently, gender was included as a control variable in the regression equation. Additionally, grade level was significantly correlated with both the independent variable and the mediating and moderating variables. Therefore, grade and gender were included as control variables in the examination of chained mediation effects and moderating effects. Gender and grade level were initially dummy-coded, with results presented in Table 4. A hierarchical regression analysis was conducted to explore the effects of negative life events, gender, grade level, basic psychological needs, anxiety, and boredom proneness on problematic online gaming use among adolescents, with findings detailed in Table 5. Initially, Equations 1 and 2 demonstrated that negative life events, gender, and anxiety significantly and positively predicted problematic online gaming use, while basic psychological needs significantly and negatively predicted it. Subsequently, upon incorporating boredom proneness in Equation 3, the predictive role of basic psychological needs became non-significant, yet the grade level emerged as a significant positive predictor of problematic online gaming use. Hypothesis 1 was thus confirmed.

**Table 4:** Virtualization treatment of control variables.

Independent variable		Assignment method		
Gender				
Gender 1		Male = 1	Female = 0	
Grade				
Grade 1	Freshman = 1	Sophomore = 0	Junior = 0	Senior = 0
Grade 2	Freshman = 0	Sophomore = 1	Junior = 0	Senior = 0
Grade 3	Freshman = 0	Sophomore = 0	Junior = 1	Senior = 0

**Table 5:** Hierarchical regression analysis.

Predictive factors	Equation 1		Equation 2		Equation 3	
	*β*	*t*	*β*	*t*	*β*	*t*
Gender1	0.18	5.59^***^	0.17	5.42^***^	0.19	6.22^***^
NLE	0.24	7.47^***^	0.10	2.68^**^	0.08	2.20^*^
BPN			−0.15	−4.14^***^	0.01	0.18
Anxiety			0.16	3.88^***^	0.11	2.88^**^
BP					0.26	6.12^***^
R^2^	0.10		0.14			
F	48.44^***^		37.68^***^		38.90^***^	

NLE, Negative life events; BPN, Basic psychological needs; BP, Boredom proneness.

### Chain mediation analysis

Negative life events significantly and negatively predicted basic psychological needs (*β* = −0.18, SE = 0.02, *t* = −10.97, *p* < 0.001), and significantly and positively predicted anxiety (*β* = 0.08, SE = 0.01, *t* = 15.65, *p* < 0.001) and problematic online gaming use (*β* = 0.01, SE = 0.00, *t* = 2.69, *p* < 0.01). Basic psychological needs significantly and negatively predicted both anxiety (*β* = −0.10, SE = 0.01, *t* = −10.54, *p* < 0.001) and problematic online gaming use (*β* = −0.02, SE = 0.01, *t* = −4.15, *p* < 0.001), while anxiety significantly and positively predicted problematic online gaming use (*β* = 0.07, SE = 0.02, *t* = 3.91, *p* < 0.001). The results of the mediation effect test, presented in Table 6, indicate that both basic psychological needs and anxiety serve as separate mediators between negative life events and problematic online gaming use, and they also exert a significant serial mediating effect. Hypotheses 2 and 3 are thus supported.

**Table 6:** Diagram of mediation effects, direct effects, and total effects decomposition.

Path	Effect	BoostSE	95%CI		Effect proportion
			Lower bound	Upper bound	
NLE → POGU	0.008	0.003	0.002	0.014	42.11%
NLE → BPN → POGU	0.004	0.001	0.002	0.006	21.05%
NLE → Anxiety→POGU	0.005	0.001	0.003	0.008	26. 32%
NLE → BPN → Anxiety→POGU	0.001	0.000	0.001	0.002	5.26%
Total indirect effect	0.011	0.002	0.007	0.014	57.89%
Total effect	0.019	0.003	0.014	0.024	

NLE, Negative life events; POGU, problematic online gaming use; BPN, Basic psychological needs; BP, Boredom proneness.

### Moderated serial mediation analysis

After standardizing the variables to examine the moderating effect of boredom proneness, with results detailed in Table 7. The interaction term between negative life events and boredom proneness significantly predicted basic psychological needs, anxiety, and problematic online gaming use, indicating that boredom proneness significantly moderated the impact of negative life events on these three variables, thereby validating Hypothesis 3.

**Table 7:** Test of moderated serial mediation effects.

	Variable			The results of the regression equation and its significance		
Result variable	Predictive variable	*R^2^*	*β*	*t*	95%*CI* Lower Bound	95%*CI* Upper Bound
BPN	NLE			−4.64^***^	−0.09	−0.04
	BP			−23.94^***^	−10.15	−8.61
	Gender 1			−3.17^**^	−3.74	−0.88
	Grade 1			0.83	−5.34	9.68
	Grade 2			0.04	−8.67	8.56
	Grade 3			0.27	−4.04	9.69
	NLE × BP	0.02	0.06	5.19^***^	0.04	0.08
Anxiety	NLE			14.31^***^	0.06	0.08
	BPN			−5. 69^***^	−0.10	−0.05
	Gender 1			1.07	−0.24	0.82
	Grade 1			−3.23^**^	−7.56	−1.23
	Grade 2			−2.27^*^	−6.55	−0.80
	Grade 3			−2.44^*^	−6.47	−0.79
	BP			5.28^***^	0.62	1.35
	NLE × BP	0.01	0.00	4.68^***^	0.01	0.03
POGU	NLE			2.17^*^	0.00	0.01
	BPN			−0.19	−0.01	0.01
	Gender 1			6.12^***^	0.59	1.15
	Grade 1			2.16^*^	0.14	2.77
	Grade 2			2.10^*^	0.08	2.34
	Grade 3			2.24^*^	0.34	3.07
	Anxiety			2.53^*^	0.01	0.08
	BP			6.15^***^	0.42	0.81
	NLE × BP	0.00	0.00	1.91^+^	0.00	0.01

NLE, Negative life events; POG, problematic online gaming use; BPN, Basic psychological needs; BP, Boredom proneness. ^+^: marginally significant.

**Figure 2:**
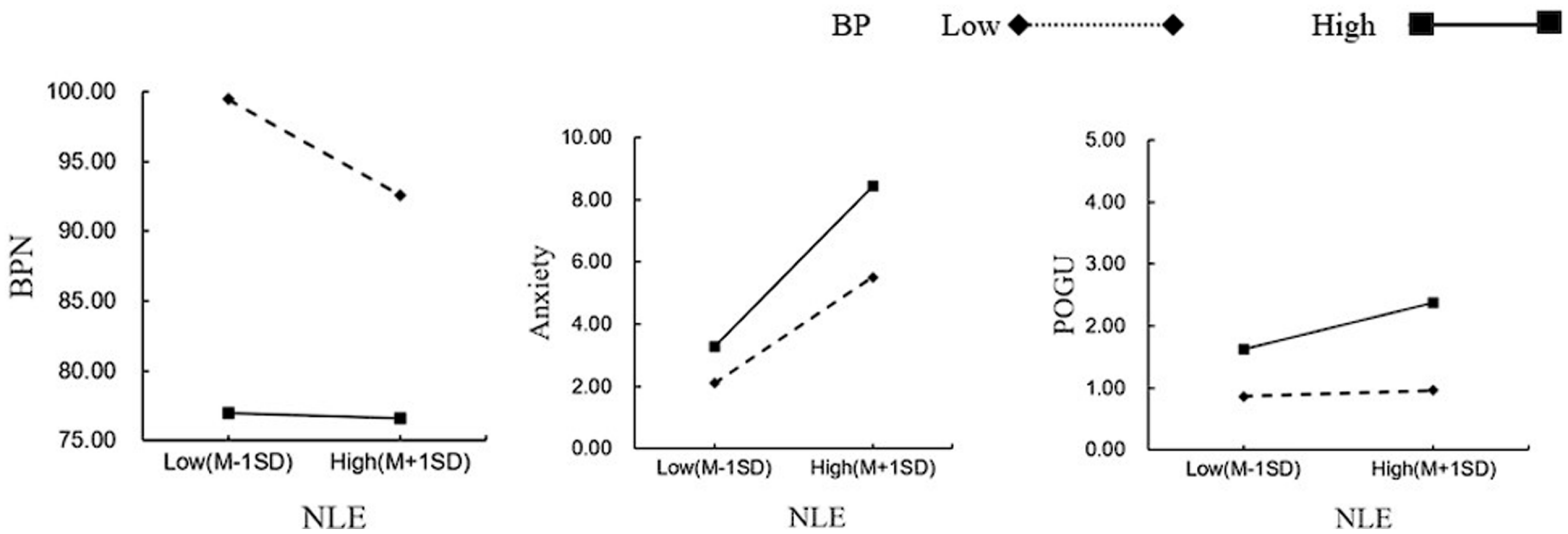
The moderating role of boredom proneness in the relationship between basic psychological needs, anxiety, and problematic online gaming use.

Standardized boredom propensity was divided into high and low groups based on one standard deviation above and below the mean, and simple slope analysis was conducted to explore the substance of the moderating effect (see Figure 2). Firstly, under the condition of high boredom propensity, the independent mediating role of anxiety is significant, *β*_anxiety_ = 0.0041, 95%CI [0.0009, 0.0076]; the independent mediating role of basic psychological needs is not significant, *β*_psychological needs_ = 0.0000, 95%CI [−0.0002, 0.0002]; the chained mediating role of basic psychological needs and anxiety is not significant, *β*_psychological needs—anxiety_ = 0.0000, 95%CI [−0.0001, 0.00001]. Under the condition of low boredom propensity, the separate mediating role of basic psychological needs is enhanced, β_psychological needs_ = 0.0001, 95%CI [−0.0014, 0.0018]; the chained mediating role of basic psychological needs and anxiety is enhanced, β_psychological needs—anxiety_ = 0.0004, 95%CI [0.0001, 0.0008]; the separate mediating role of anxiety is diminished, β_anxiety_ = 0.0023, 95%CI [0.0001, 0.0044]. Simple slope analysis indicates that under high boredom propensity, negative life events do not significantly predict basic psychological needs (*β* = −0.002, SE = 0.017, *t* = −0.140, *p* = 0.888), but significantly predict anxiety and internet gaming addiction (*β* = 0.092, SE = 0.006, *t* = 14.545, *p* < 0.001; *β* = 0.011, SE = 0.004, *t* = 2.902, *p* = 0.004). Under low boredom propensity, negative life events significantly predict basic psychological needs in a negative direction (*β* = −0.122, SE = 0.018, *t* = −6.693, *p* < 0.001), the positive predictive effect on anxiety is diminished (*β* = 0.051, SE = 0.007, *t* = 7.460, *p* < 0.001), and there is no significant predictive effect on internet gaming addiction (*β* = 0.002, SE = 0.004, *t* = 0.528, *p* = 0.598). Secondly, this suggests that the moderated chained mediating effect is established.

## Discussion

This study examines the impact of negative life events on college students’ problematic online gaming use, focusing on the serial mediating role of basic psychological needs and anxiety, as well as the moderating effect of boredom proneness. It addresses three key questions: whether negative life events affect college students’ problematic online gaming use, what the underlying mechanisms are, and what boundary conditions exist.

### Negative life events and college students’ problematic online gaming use

Negative life events significantly and positively predict problematic online gaming use among college students, aligning with previous research (Shi et al., 2021; Torres-Rodríguez et al., 2018), thereby confirming Hypothesis 1. Internet addiction serves as a coping strategy for adolescents facing stressful life events, as immersion in internet use can provide temporary relief from pressures exerted by family and school, offering psychological liberation (Li et al., 2022). When confronted with negative life events, an individual’s internal equilibrium is disrupted, necessitating a means to alleviate or release stress in pursuit of a new state of balance (Huang, 2022). This is consistent with the theory of stress relief, which posits that addicts cannot control their internet use because they seek to reduce stress through this medium, and individuals who are more sensitive to stress are more likely to develop such coping mechanisms (Kim and Davis, 2009). Internet gaming allows individuals who perceive stressful events to temporarily escape from this stress, and once they deem this behavior effective, their risk of developing problematic online gaming use significantly increases. Our results demonstrate the impact of environmental factors on individual problematic online gaming use, supporting the arguments of the I-PACE model and the Bioecological Model regarding the influence of environmental factors on individual addictive behaviors.

### The serial mediating role of basic psychological needs and anxiety between negative life events and college students’ problematic online gaming use

The findings of this study establish a serial mediation model elucidating the relationship between negative life events and problematic online gaming use among college students, detailing the mediating mechanisms of basic psychological needs and anxiety. Negative life events are negatively correlated with basic psychological needs, indicating that when individuals encounter such events, the likelihood of their psychological needs being met diminishes. As a significant mediator between negative life events and college students’ problematic online gaming use, unmet basic psychological needs negatively predict the likelihood of addiction. Research found that adverse life events, such as poor parental communication and insufficient school engagement, can make it difficult for adolescents to have their psychological needs fulfilled, increasing the risk of seeking psychological satisfaction through internet gaming (Tian et al., 2018). The Uses and Gratifications theory suggests that individuals actively and purposefully engage in internet gaming to fulfill specific psychological needs (Katz et al., 1974). This implies that those who do not have their psychological needs met in daily life are inclined to use the internet as a compensatory mechanism to cope with negative life conditions, increasing their risk of problematic online gaming use (Hong et al., 2020; Liu et al., 2016).

The study reveals that anxiety serves as a mediator in the influence of negative life events on college students’ problematic online gaming use. According to the cognitive-behavioral model, external environmental stimuli and the resulting adverse emotional states are among the factors leading to the development of addictive behaviors (Davis, 2001). Research showed that negative life events can lead to adverse emotions such as anxiety and depression in college students, with those having higher anxiety levels exhibiting lower self-esteem and self-evaluation, perceiving themselves as lacking the ability to handle stressful events (Liu et al., 2018). Consequently, when confronted with negative life events, they are more likely to perceive stress, and this maladaptive cognition can further induce internet addiction (Zhang et al., 2016; Kim and Davis, 2009), corroborating the Vulnerability-Stress model. Individuals with higher anxiety levels are more prone to perceive stress in the same stressful situations and thus are more likely to become engrossed in the internet. When college students are aware of negative life events around them, they can easily develop persistent anxiety under the interaction of these events and negative cognitions (Xin et al., 2023). If they fail to manage these negative events, their development may be hindered, and the anxiety that ensues, if alleviated through internet gaming, can lead to a tendency towards problematic online gaming use among college students.

This study identified a significant serial mediation pathway from “fulfilled basic psychological needs to anxiety” between negative life events and problematic online gaming use, confirming Hypothesis 2. When individuals experience negative life events, their basic psychological needs remain unmet, leading to anxiety in college students, which in turn drives them towards increased internet gaming behavior. This outcome aligns with the “loss-compensation” hypothesis (Gao and Chen, 2006), suggesting that when college students are subjected to negative life events, the dissatisfaction of their basic psychological needs disrupts their normal development, resulting in anxiety due to developmental hindrance. To alleviate this anxiety, they may resort to pathological compensation, thus increasing the risk of problematic online gaming use. Our successfully validated serial mediation pathway also indicates that individual emotional and cognitive components can serve as mediators for problematic online gaming use. Furthermore, we have provided a more detailed explanation of this pathway by integrating Self-Determination Theory and the Stress-Vulnerability Model.

### The moderating role of boredom proneness

The findings indicate that boredom proneness significantly moderates the impact of negative life events on basic psychological needs, anxiety, and problematic online gaming use, thereby validating Hypothesis 3. In the current society, where “involution” is prevalent, individuals with high levels of boredom proneness, when confronted with increased academic pressure and the intensification of interpersonal conflicts, may struggle to employ rational methods to promptly alleviate or transform their feelings of boredom, potentially leading to behavioral deviations, such as engaging in more gaming activities (Wang Y. et al., 2020). Individuals with high boredom proneness often harbor dissatisfaction with their life and academic status when facing academic pressures or interpersonal conflicts. This dissatisfaction results in a lack of fulfillment of their basic psychological needs, an absence of intrinsic motivation, and a perception that academic and social interactions are devoid of meaning, propelling them to use internet gaming as an escape from their wearisome emotions (Huang et al., 2020; Huang et al., 2023). Adolescents with high boredom proneness are more likely to have their cognition confined to negative contexts, and prolonged boredom can more readily draw their attention to negative stimuli, ensnaring them in a vicious cycle of negative cognitive patterns and increasing the likelihood of developing negative emotions such as depression and anxiety (Yu and Zhang, 2021; Zhu et al., 2019; Yan et al., 2021). Consequently, when individuals with high boredom proneness encounter the stress of negative life events, their basic psychological needs are even less likely to be met, the potential for anxiety is heightened, and the risk of problematic online gaming use is exacerbated. The results of this study further substantiate the Bioecological Model.

The typical characteristic of boredom proneness is low perceived control over activities. When faced with stressful events, individuals with high boredom proneness are more likely to experience a sense of loss of control, doubt their ability to cope effectively with stress, and lack the means and willingness to seek help. This leads to negative experiences in terms of belonging, competence, and autonomy needs for individuals with high boredom proneness, thereby reducing the degree to which their basic psychological needs are met. Under the interaction of stressful situations and negative personality traits, individuals are more likely to experience anxiety. The high positive feedback and low cognitive cost of internet games provide a compensatory pathway for individuals to alleviate anxiety, making it easier for them to compensate for the basic psychological needs missed due to stressful events through internet games. The I-PACE model suggests that situational factors are subjectively perceived, and the level of perceived stress is related to emotional and cognitive responses, which in turn affect subsequent cognitive processes. Internet addiction may be positively correlated with positive expectations (e.g., experiencing pleasure) and avoidance expectations (e.g., escaping reality) at the bipolar level (Brand et al., 2016), and these two types of expectations are also related to boredom proneness traits and stressful events to a certain extent.

### Research significance and limitations

#### Theoretical significance

**Figure 3:**
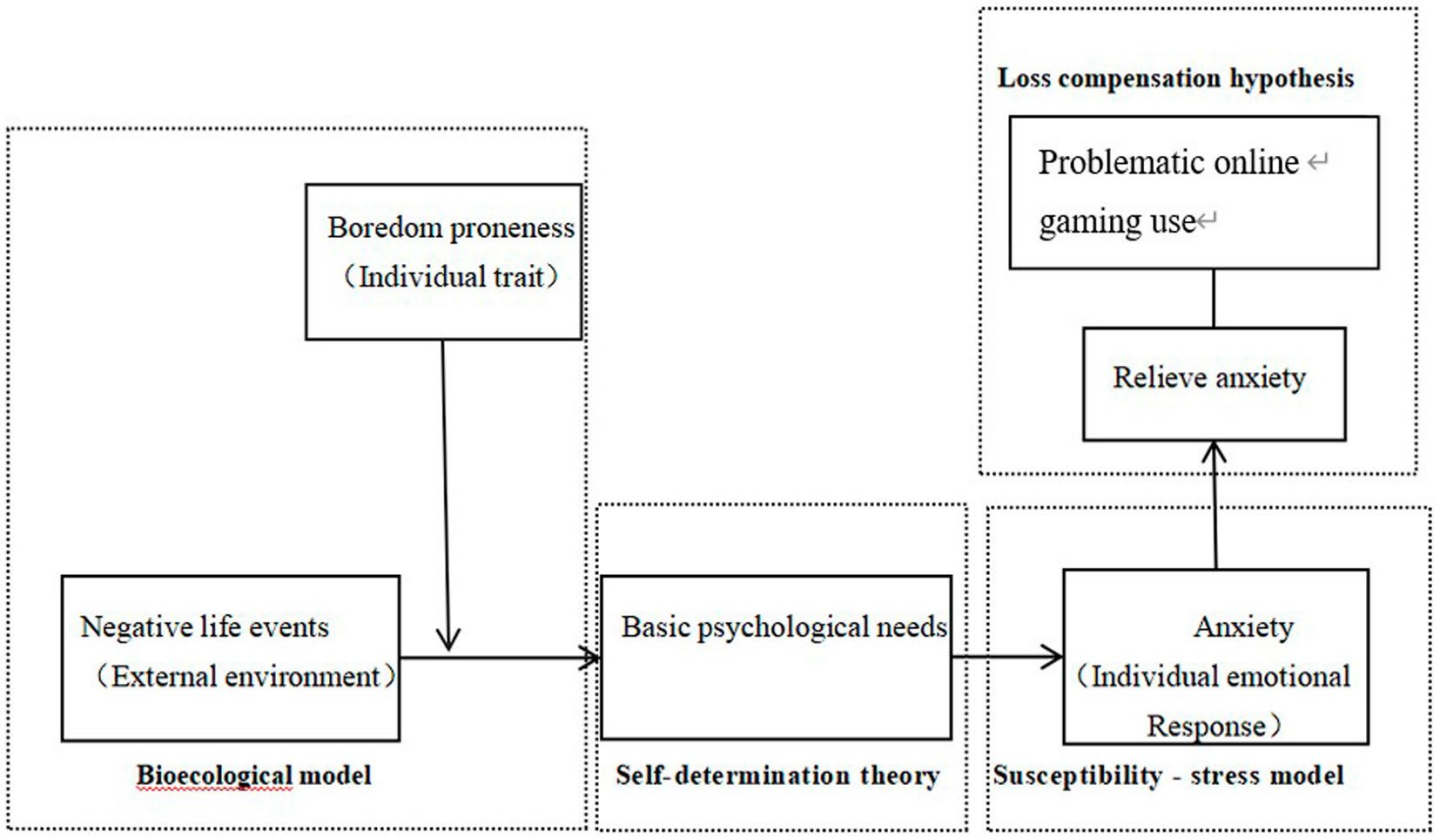
Model of the process of problematic online gaming use development based on the I-PACE framework.

This study confirms that problematic online game use is the result of the interaction of multiple factors, rather than something that can be explained by a single factor. This perspective provides deeper theoretical support for the I-PACE model. Previous research has largely been conducted based on the I-PACE model. Additionally, it included only personality traits or cognitive factors, which were relatively isolated and lacked discussion of the interplay between variables. For instance, Jhone et al. (2021) used the I-PACE model as a foundation to discuss the impact of adverse childhood experiences on middle school students’ problematic online gaming use and the mediating role of stress. Our study further enriches the content of the I-PACE model, validating it as a framework to examine hypotheses about the interactions between specific characteristics, including personality traits, cognitive and affective processes. This study adopts the I-PACE model as a framework and incorporates Self-Determination Theory, the Vulnerability-Stress Model, and the Bioecological Model to thoroughly discuss the influence of each variable on problematic online gaming use and the different theoretical foundations at each stage of the formation process of individual problematic online gaming use. The study has concretized the variables of “personality,” “cognition,” and “emotion” proposed by the I-PACE model, thereby further enhancing the operability and explanatory power of the I-PACE model in explaining the psychological mechanisms underlying problematic online game use. Therefore, this research, starting from the formation process of internet addiction, constructs a model of the formation process of adolescent problematic online gaming use within the I-PACE framework (Figure 3).

This study, in conjunction with the “Bioecological Model,” confirms that “negative life events” act as an independent variable that plays both direct and indirect roles in problematic online gaming. Although the I-PACE model takes into account external inducements, it does not delve into the impact of negative life events on behavioral addictions. By integrating the Bioecological Model, we can more comprehensively understand how external environments or events interact with individual characteristics to influence behavioral addictions. This expands our understanding of the external inducements of behavioral addictions, extending beyond direct online stimuli such as social media and games to include a broader range of life events. This contributes to a more holistic comprehension of the causes and mechanisms of behavioral addictions.

### Practical significance

Firstly, we should focus on the impact of negative life events on college students, pay attention to the stress brought about by the negative life events they face, and provide help from their perspective to prevent the dissatisfaction of psychological needs and anxiety arising from “boring tendencies” and negative personality traits when facing stress. This can break the vicious cycle of seeking compensation from the internet and prevent the formation of problematic online gaming use. Secondly, educators can help students gain interpersonal support from teachers and friends through school activities. Schools can also offer specialized mental health education courses, teaching students various self-regulation methods, such as mindfulness therapy, art therapy, etc., to change their boredom and generate pleasant emotions in real life, fulfill or compensate for the students’ psychological needs, alleviate anxiety, and thus promote the development of students. When students’ psychological needs are met in real life, even if they use the internet, it can reduce the likelihood of them becoming addicted to the internet instead of seeking compensation online.

### Limitations

The study also has certain limitations. First, the sample group of this study comes from the same university, and the results may not be generalizable to other populations. Future research could enhance sample representativeness to improve the ecological validity of the study. Second, the cross-sectional research design limits the inference of causality between variables, and future studies could conduct longitudinal research-based serial mediation effect.

## Conclusion

The results of the study showed that (1) Negative life events and anxiety significantly and positively predict problematic online gaming use, while basic psychological needs significantly and negatively predict problematic online gaming use. (2) Both basic psychological needs and anxiety exert independent mediating effects on the relationship between negative life events and problematic online gaming use, and they also operate in a serial mediating capacity. (3) Boredom proneness significantly moderates the impact of negative life events on basic psychological needs, anxiety, and problematic online gaming use.

## Data Availability

The raw data supporting the conclusions of this article will be made available by the authors, without undue reservation.
